# Supplementary material for: Clinical Outcomes in COVID-19 Patients Treated with Immunotherapy
Source: Cancers (Basel). 2022 Dec 1;14(23):5954. doi: 10.3390/cancers14235954 (PMC9735726; doi:10.3390/cancers14235954)
Supplement: Supplementary file 1 [file cancers-14-05954-s001.zip › cancers-1928813-supplementary.pdf]

cancers

MDPI

Supplementary Materials: Clinical Outcomes in COVID-19 Patients Treated with Immunotherapy

Haris Hatic, Kristine R. Hearld, Devika Das and Jessy Deshane

Table S1. Characteristics of the Sample, COVID Infected, Just Lung Cancer (n = 40).

Patient Characteristic		Treatment		
	Sample	Chemotherapy (n = 20)	Immunotherapy (n = 20)	t/X²
**Socio-Demographics**				
Age (M/SD)	67.13 (9.59)	70 (2.33)	64.25 (7.92)	1.963 *
Sex				
Female	17 (42.50%)	9 (45.00%)	8 (40.00%)	
Male	23 (57.50%)	11 (55.00%)	12 (60.00%)	0.102
Race/Ethnicity				
Black	14 (35.00%)	7 (35.00%)	7 (35.00%)	
White	25 (62.50%)	13 (65.00%)	12 (60.00%)	
Asian/Latinx/Other	1 (2.50%)	0 (0.00%)	1 (5.00%)	1.040
**Clinical Factors**				
Comorbidities				
0	0 (0.00%)	0 (0.00%)	0 (0.00%)	
1–2	1 (2.50%)	0 (0.00%)	1 (5.00%)	
3–5	26 (60.00%)	14 (70.00%)	12 (60.00%)	
>5	13 (32.50%)	6 (30.00%)	7 (35.00%)	1.231
ECOG				
0–1	33 (82.50%)	17 (85.00%)	16 (80.00%)	
2–4	7 (17.50%)	3 (15.00%)	4 (20.00%)	0.173
Symptoms at COVID Diagnosis				
Fever	6 (15.00%)	5 (25.00%)	1 (16.67%)	3.137 *
Cough	11 (27.50%)	6 (30.00%)	5 (25.00%)	0.125
Dyspnea	18 (45.00%)	12 (60.00%)	6 (30.00%)	3.636 *
PD-L1 Expression (M/SD)	24 (34.91)	25.3 (8.66)	22.7 (31.62)	0.233
Immunotherapy				
PD-1/PD-L1		--	19 (95.00%)	
CTLA-4		--	--	
Combined		--	1 (5.00%)	
Admission				
Home	27 (67.50%)	16 (80.00%)	11 (55.00%)	
Floor	9 (22.50%)	3 (15.00%)	6 (30.00%)	
Intensive Care	4 (10.00%)	1 (5.00%)	3 (15.00%)	2.926
Oxygen Use	11 (27.00%)	5 (25.00%)	6 (30.00%)	0.125
Mechanical Ventilation	1 (2.50%)	0 (0.00%)	1 (5.00%)	1.026
Treatment				
Steroids	11 (27.50%)	4 (20.00%)	7 (35.00%)	1.129
Antiviral	6 (15.00%)	3 (15.00%)	3 (15.00%)	0.000
Antibiotics	12 (30.00%)	7 (35.00%)	5 (25.00%)	0.476
Mortality	7 (17.50%)	5 (25.00%)	2 (10.00%)	1.558

*$p < 0.05$.

Table S2. Characteristics of the Sample, COVID Infected, all Cancers (*n* = 121).

Patient Characteristic		Treatment		
	Sample	Chemotherapy (*n* = 60)	Immunotherapy (*n* = 61)	t/X^2
**Socio-Demographics**				
Cancer				
Cold	45/37.19%	25/41.67%	20/32.79%	
Warm	74/61.16%	34/56.67%	40/65.57%	
Neither	2/1.65%	1/1.67%	1/1.64%	1.034

There is not a statistically significant association between cancers (cold, warm, neither) and treatment ($p = 0.596$).

Table S3. Association between Clinical Factors and Smoking Status, COVID Infected (*n* = 121).

Patient Characteristic	Not a Smoker	Current Smoker	Former Smoker	t/X^2
Cancers				
Cold	16/47.06%	10/50.00%	19/28.36%	
Warm	17/50.00%	9/45.00%	48/71.64%	
Neither	1/2.94%	1/5.00%	0.00%	8.732 *

*$p < 0.05$; There is a statistically significant association between cancers (cold, warm, neither) and smoking status ($p = 0.029$).

Table S4. Association between Clinical Factors and Covid Vaccine Status, COVID Infected (*n* = 121).

Patient Characteristic	Covid Vaccine	No Covid Vaccine	t/X^2
Cancers			
Cold	21/41.18%	24/34.29%	
Warm	30/58.82%	44/62.86%	
Neither	0/0.00%	22.86%	1.912

There is not a statistically significant association between cancers (cold, warm, neither) and COVID vaccine ($p = 0.384$).

Table S5. Risk Factors of Admission, ICU, and Mortality, COVID Infected (*n* = 121).

Patient Characteristic	Admission	ICU	Death
Cancers			
Cold	24/34.29%	3/33.33%	36/36.73%
Warm	45/64.29%	6/66.67%	60/61.22%
Neither	1/1.43%	0/0.00%	2/2.04%

There is not a statistically significant association between cancers (cold, warm, neither) and admission, ICU, or death. For Admission: $p = 0.707$, IUC: $p = 0.883$, death: $p = 0.779$.

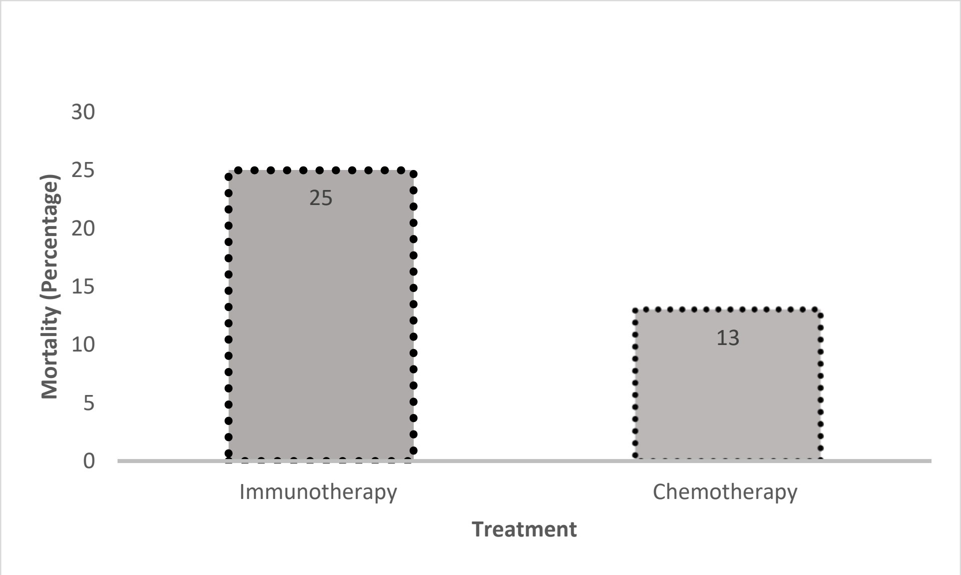

Figure S1. Mortality of COVID Infected Patients (n = 121).
